# Supplementary material for: Development and validation of a multiplex real-time qPCR assay using GMP-grade reagents for leprosy diagnosis
Source: PLoS Negl Trop Dis. 2022 Feb 18;16(2):e0009850. doi: 10.1371/journal.pntd.0009850 (PMC8893668; doi:10.1371/journal.pntd.0009850)
Supplement: S1 Text — (DOCX) [file pntd.0009850.s001.docx]

**S1 Text.** Sequences of the synthetic DNA template control (“gBlocks”).

CAGGCAGCAGTATCGTGTTAGTGAACAGTGCATCGATGATCCGGCCGTCGGCGGCACATACGGCAACCTTCTAGCGCAGGGCGCATGTCTTGTGGTGGAAAGCTTTTTGCGGTGCAGGATGGGCCCGCGGCCTATCAGCTTGTTGGTGGGGTGGGCCTAGAAACTGCGAATGGCTCATTAAATCAGTTATGGTTCCTTTGGTCGCTCGCTCCTCTCCTACTTGGATAACTGTGGTAATTCTAGAGCTAATACATGCCGACGGGCTA
