# Supplementary figures and images for: Development and validation of a multiplex real-time qPCR assay using GMP-grade reagents for leprosy diagnosis
Source: PLoS Negl Trop Dis. 2022 Feb 18;16(2):e0009850. doi: 10.1371/journal.pntd.0009850 (PMC8893668; doi:10.1371/journal.pntd.0009850)

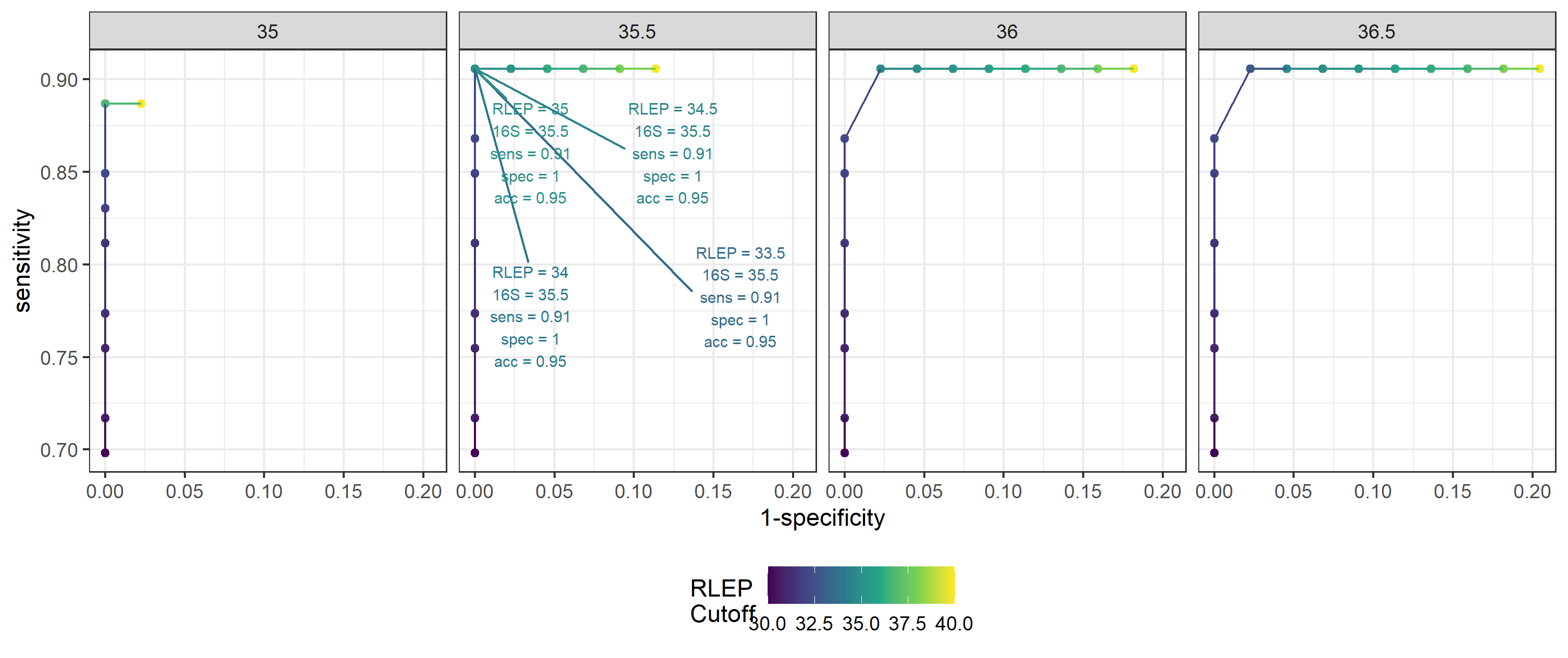

Supplement: S1 Fig — Different combinations of cutoff values for 16S rRNA (panels) and RLEP (color scale) were tested on a patient panel (n = 97). For each combination of cutoff values, the sensitivity and specificity were calculated and plotted as ROC curves. Here, only Cp cutoff values for 16S rRNA between 35 and 36.5 are shown. The combinations resulting in a specificity of 1 and the highest sensitivity for each condition are annotated. (TIFF) [file pntd.0009850.s002.tiff]
