# Supplementary material for: Development and validation of a multiplex real-time qPCR assay using GMP-grade reagents for leprosy diagnosis
Source: PLoS Negl Trop Dis. 2022 Feb 18;16(2):e0009850. doi: 10.1371/journal.pntd.0009850 (PMC8893668; doi:10.1371/journal.pntd.0009850)
Supplement: Attachment — Submitted filename: Responses to the review comments.docx [file pntd.0009850.s009.docx]

**Dear Editor,**

**In reference to the paper “Development and validation of a multiplex real-time qPCR assay using GMP-grade reagents for leprosy diagnosis” by Manta and Jacomasso et al, and Manuscript reference number: PNTD-D-21-01417, we would like to thank the reviewers for their overall assessment of our paper with criticisms that certainly will improve the final version. Please find attached a version containing the tracked changes as well as a clean version of the manuscript. We answered point-by-point all the questions that can be seen below in red. We hope that this version is now suitable for publication in PLOS Neglected and Tropical Diseases.**

**Cordially,**

**Milton Moraes and Alexandre Costa**

**Responses to the review comments**

**Reviewer #1:**

**1. The article by Alexandre DT Costa et al. describes the development of multiplex real-time qPCR for the early diagnosis of leprosy. The evaluation of the diagnostic test using real time qPCR with clinical specimens of leprosy as well as other dermatological specimens has shown good performance in terms of sensitivity and specificity.**

**We are thankful for the nice overall assessment of our paper.**

**2. Major concern is Figure 6, which shows the distribution of Cp values of multiplex real time qPCR. It is questionable whether multiplex qPCR (using both RLEP and 16sRNA) rather than detection of RLEP alone, is necessary for leprosy diagnosis especially in a clinical setting. For the detection of M. leprae, RLEP is generally more sensitive than 16S rRNA in a PCR reaction (well accepted observation), but 16S rRNA may be show higher specificity. To prove that multiplex qPCR (using both RLEP and 16sRNA) is more efficient, direct comparison and statistical evaluation of real time qPCR using RLEP alone and both RLEP and 16sRNA is needed**

**The reviewer raises a valid point. The same figure 6 (which became figure 5 after review) shows that RLEP is detected in six samples of “other skin diseases” (open circles) out of 44, as the correspondent points appear to the left of the RLEP cutoff line. In the case where only RLEP was used for classification, these samples would fall under the category of false positives. Patients diagnosed in this way would undergo a long and taxing treatment without actually carrying *M. leprae*. The multiplexed qPCR using 16S rRNA allows for the screening of potential false positives (which, in the cohort we used, clustered above the 16S cutoff), therefore increasing the specificity of the test. As for the statistical analyses requested, we took a conservative approach and considered samples in the “equivocal” quadrant (positive for RLEP and negative for 16S rRNA) as negative samples when calculating Sensitivity/Specificity. Thus, a statistical comparison between RLEP alone and RLEP/16S multiplex would not show the nuance of the added benefit of 16S. We believe that the figure and the associated data in the supplement contain all the information needed to answer the reviewer’s question. We agree, however, that this point is not very clear in the text. Sentences in lines 446-450 in the Results section were rewritten to convey this message and now reads : “Taken together, these results demonstrate that the multiplexed reaction for *M. leprae* RLEP and 16S rRNA is able to classify samples precisely, by combining the strengths of each molecular target and improving on their use in isolation. This setup is also able to flag samples with low bacilli burden for further monitoring, avoiding unnecessary treatment of uninfected patients and proper follow up of *M. leprae* carriers.**”

**A sentence : “ Samples that fall under the “Equivocal” category were considered to be negative when calculating the diagnostic sensitivity, specificity and the predictive values. These parameters were calculated according to Altman and Bland (1994).” was also added to lines 274-277 of the Data Analyses and Statistics section to clarify how the “equivocal” samples were treated when calculating the sensitivity and specificity of the test.**

**Also, a comparison between RLEP and RLEP+16S is shown in the table below with the diagnostic performance parameters determined for the RLEP reaction alone:**

**type	tp	tn	fp	fn	n	sens	spec	PPV	NPV	acc**

**Multibacillary	35	38	6	0	79	1.00	0.86	0.85	1.00	0.92**

**Paucibacillary	17	38	6	1	62	0.94	0.86	0.74	0.97	0.89**

**Combined	52	38	6	1	97	0.98	0.86	0.90	0.97	0.93**

**This data should be compared with the parameters determined for the test as described in the manuscript (RLEP +16S):**

**type	tp	tn	fp	fn	n	sens	spec	PPV	NPV	acc**

**Multibacillary	33	44	0	2	79	0.94	1	1	0.96	0.97**

**Paucibacillary	15	44	0	3	62	0.83	1	1	0.94	0.95**

**Combined	48	44	0	5	97	0.91	1	1	0.90	0.95**

**We added this last Table as supplementary table 5 to also facilitate readers assessment.**

**3. The accuracy, reproducibility as well as the stability of the multiplex qPCR should be evaluated using the DNA samples from the biopsies in addition to the synthetic DNA template.**

**We respectfully disagree with the reviewer. Accuracy, reproducibility, and stability tests are designed to evaluate the reagents and their presentation format, not the templates used in the reactions. Specifically, the stability test evaluates if one of the key performance indicators, the limit of detection (LOD), remains constant using the provided reagents. If the LOD is maintained throughout the study period, one can expect that all other performance indicators will remain stable as well.**

**Furthermore, once extracted using high quality reagents and properly stored, the DNA template has no relationship with the reaction’s performance regarding stability, accuracy, or reproducibility of a diagnostic test. We agree that the quality of the DNA template, and thus its stability, might affect the test’s sensitivity and specificity, but DNA sample quantity and quality was the focus of a previous publication of our group (Manta et al 2020, 10.1371/journal.pntd.0008325) and is not addressed in the present manuscript.**

**Minor comments:**

**4.Table 1 shows the characteristics of the patients including both leprosy and other dermatological diseases. Criteria for the selection of the 2nd panel has to be described in the Table or Methods. PN describes Pure Neural cases of leprosy but none seem to be involved in the study.**

**Thanks for the comments. We removed the phrase that referred to nerve samples as they were not actually analyzed in this work and expanded the section “clinical samples” on methods (lines 164-166) to better explain the selection of samples that now reads: “The samples were included according to patient enrollment consecutively and are representative of the period between 2010 and 2018”.**

**Reviewer #2:**

**Reviewer’s comments on the manuscript entitled " Development and validation of a multiplex real-time qPCR assay using GMP-grade reagents for leprosy diagnosis.”**

**Brief summary: The authors have developed a multiplex qPCR targeting 16S and RLEP genomic regions of M. leprae and have included human 18S rRNA sequences as an inbuilt control of successful DNA extraction from patient clinical samples. They have validated their assay in test and validation set of samples and have reported excellent sensitivity and specificity. This is a very important development and work is carried out very well. A few query are shown below. In addition, a few changes may be incorporated to improve the manuscript further.**

**We thanks the reviewer for his/her insightful comments of our paper.**

**Major comments:**

**1. As identified by the authors, there are fewer samples of paucibacillary patients. As the sensitivity of a test varies significantly based on the number of paucibacillary samples included in the analysis, the authors should present their findings about the test performance parameters separately for the paucibacillary and multibacillary samples.**

**Please see the answer for the next question.**

**2. Positive Predictive Value (PPV) and Negative Predictive Values (PPV) of the qPCR assay can be mentioned while writing the Sensitivity, Specificity and Accuracy, preferably separately for the paucibacillary and multibacillary cases.**

**We agree with the reviewer and included the parameters (including PPV and NPV) among the group of Multibacillary and Paucibacillary. We added a supplementary table 5 (please see below) to the manuscript to summarize the results in a clearer form.**

**type	tp	tn	fp	fn	n	sens	espec	PPV	NPV	acc**

**Multibacillary	33	44	0	2	79	0.94	1	1	0.96	0.97**

**Paucibacillary	15	44	0	3	62	0.83	1	1	0.94	0.95**

**Combined	48	44	0	5	97	0.91	1	1	0.90	0.95**

**3. If there was a selection criteria of the patients (whether consecutive samples, or from a specified duration), please mention it after the sentence in line# 160.**

**Thank you for raising this point. The selection criteria for the 2nd panel was similar to the first. That is, diagnosis was made on the basis of histopathological and clinical findings irrespective of lesion or slit-skin smear bacilli index. Also, the sampling scheme was consecutive according to the clinic routine. The samples used in this study are representative from the period between 2010-2018 This information was added in the Methods section under the “clinical samples” subsection.**

**4. The Table 1 (on page 10), can be updated with the qPCR results as one more column, which will enable the reader to identify the qPCR positivity trends for the PB and MB cases; and its correlation with the Bacterial Index (written as “Bacterial load” in Table 1). Indeed, “Bacterial load” can be replaced with B.I. to be more accurate.**

**We agree with the reviewer regarding showing the parameters for Multibacillary and Paucibacillary clinical forms in separate lines, and therefore we included these results in a new supplementary table 5. The correlation between bacterial index and qPCR results is shown in figure 5. “Bacterial load” was replaced by “Bacterial index” throughout the manuscript, as requested.**

**5. Line 398: Regarding the samples in the “Equivocal” quadrant, has there been a re-testing of the same DNA samples? This is important since as a new sample is generally difficult to obtain in most settings.**

**As the reviewer points out, obtaining samples is generally difficult. Therefore, our study was performed using a convenience sample of previously collected samples, which hampers our ability to re-test those specific samples in the “Equivocal” quadrant. However, should this result appear in real life, our recommendation is to re-evaluate the patient with a new sample. Albeit difficult, that is the recommendation.**

**6. Lines 473-477: The sentence is not clear “Combining multiple primers and probes did not affect the efficiency of the triplex qPCR in comparison to the corresponding singleplex reactions used in Martinez et al. (16), who evaluated the independent detection of 16S and RLEP using the same primers and probes and obtained 0.91 and 0.51 for sensitivity and 0.73 and 1 for specificity, respectively. Does it mean, the sensitivity of 16S rRNA based detection was higher (0.91) than that of RLEP (0.51), or is it listed in reverse order? Authors may check this statement again and reword/correct it as necessary.**

**We are grateful for the reviewer for spotting this mistake that is now corrected in the updated text. Indeed, The correctly is that for RLEP, the specificity was 73% and sensitivity was 91% and for 16S, the specificity was 100% and sensitivity was 51%. Now in lines 488-490, the text reads: “They evaluated the independent detection of 16S rRNA and RLEP using the same primers and probes and obtained 0.91 and 0.51 for sensitivity and 0.73 and 1 for specificity, respectively”.**

**7. Are these primers described for 16S rRNA specific for M. leprae, and would they give any results with M. lepromatosis also? Since M. lepromatosis is also a causative agent in a significant number of cases, specially in Mexico and other countries, it would be good that this information is included in the paper. Authors can state this with experimental data or can add a small paragraph with the bioinformatic analysis of the corresponding sequences regarding the level of matching of the primers with M. lepromatosis sequences.**

**We appreciate the reviewer for pointing out this detail. According to BLAST and in silico PCRs, the RLEP primers are specific to *M. leprae* without any complete (whole oligonucleotide) identical matching to *M. lepromatosis* or any other mycobacteria. However, 16S rRNA primers completely match *M. lepromatosis* genome sequences, except for a single base mismatch in the probe sequence. We inserted this comment in the Quantitative PCR subsection in Methods.**

**We also tested *M. lepromatosis* DNA with the newly developed reactions. Although we didn't show in the manuscript because there were too few samples, the results confirm the prediction by the in silico analyses as seen in the images below.**

**DNA: M. lepromatosis; Host: red squirrel**

**8. This article can be shortened and a lot of material can be shifted to supplementary Information.**

**For this purpose, we removed the figure 5 from the text and placed it as a supplement section.**

**9. Line 293-294: Please mention how the “450 fg of DNA (ca. 126 M. leprae genomes) is converted? Is it based on the microscopy based estimation of the bacterial counts or based on the Qubit/Nanodrop based estimation of the DNA quantity converted into bacterial DNA copy number? What was the source of the DNA sample and how contribution of the host DNA nullified in the DNA preparation, if the standard DNA was from an infected animal tissue?**

**The mass was measured after quantification with a NanoDrop and converted to genome-equivalents using the size of the genome of *M. leprae* (roughly 3.3 Mbp according to NCBI genomes). The DNA copy number was calculated using a tool available at the Thermo Scientific website:**

**(**[**https://www.thermofisher.com/br/en/home/brands/thermo-scientific/molecular-biology/molecular-biology-learning-center/molecular-biology-resource-library/thermo-scientific-web-tools/dna-copy-number-calculator.html**](https://www.thermofisher.com/br/en/home/brands/thermo-scientific/molecular-biology/molecular-biology-learning-center/molecular-biology-resource-library/thermo-scientific-web-tools/dna-copy-number-calculator.html)**)**

**The protocol for purification of *M. leprae* cells ensures that the animal DNA (along with cell debris) is removed before lysing the *M. leprae* cells to extract its DNA. After the extraction, qPCR for 18S (a multicopy target in mammals genome) is performed as a quality check and only reveals traces of animal material in the starting DNA extract. Thus, we conclude that the quantity of animal DNA in our samples is not likely to cause a large error in the estimation in the number of genome-equivalents of *M. leprae*. Moreover, if the amount of DNA in the final extract remains considerable, we would be overestimating the number of genome-equivalents. This would mean that the sensitivity of our test could detect even lower amounts of *M. leprae* in the sample than what is stated in the manuscript.**

**To clarify this point in the text, we added a sentence in the Materials and Methods section “(approximately 1.4 x 10^-1^ to 1.4 x 10^6^ genome-equivalents/reaction, considering the *M. leprae* genome size to be 3.3 Mbp),” in lines 236-237 to better describe this procedure.**

**10. Line 229: “1.83 to 1.83 x 10E+07 copies/reaction”. Can it be represented as “1.83X10E+0 to 1.83 x 10E+7) to imply the intended meaning more clearly?**

**Thanks. We've changed the text to make it clearer.**

**11. Line 274-276: Cp (Crossing point) values for each target were set to intercept the positive controls and avoid the negative ones, being established as follows: 0.2 for RLEP, 0.15 for 16S rRNA, and 0.16 for 18S rRNA”. Are these values static between different experiment or can vary between different experiments? Is the background adjustment necessary for each experiment?**

**These values were determined for the 7500 Real-Time PCR system (Applied Biosystems/Thermo Scientific), and should work for every run using instruments of this model. For other qPCR instruments, the positive and negative controls should be used as a reference when determining the threshold point. Baseline/background adjustment should be adjusted automatically by the operating software.**

**This information is described at the end of the first paragraph of Results.**

**Minor comments**

**12.	One more round of careful proofreading of the manuscript will be helpful, to find out some minor typos such as “16SrRNA” “18SrRNA” (space).**

**We agree, thanks**

**13. A few corrections in language are needed, for example: the sentence “Bacterial load is show as ….” . Likewise, in line 310 “ associated with skin diseases such Leishmania …:**

**We made the corrections accordingly and also check the text.**

**14. Line 301 (tom and Bottom): Top and bottom.**

**Thanks, changed.**
